# Oleic acid induces apoptosis and autophagy in the treatment of Tongue Squamous cell carcinomas

**DOI:** 10.1038/s41598-017-11842-5

**Published:** 2017-09-12

**Authors:** Lin Jiang, Wei Wang, Qianting He, Yuan Wu, Zhiyuan Lu, Jingjing Sun, Zhonghua Liu, Yisen Shao, Anxun Wang

**Affiliations:** 1 Department of Oral and Maxillofacial Surgery, First Affiliated Hospital, Sun Yat-Sen University, Guangzhou, Guangdong, 510080 China; 2 Department of Oral and Maxillofacial Surgery, Affiliated Hospital of Jiangxi University of Traditional Chinese Medicine, Nanchang, Jiangxi Province 330006 China; 3 School of Stomatology, Nanchang University, Nanchang, Jiangxi Province 330006 China

## Abstract

Oleic acid (OA), a main ingredient of Brucea javanica oil (BJO), is widely known to have anticancer effects in many tumors. In this study, we investigated the anticancer effect of OA and its mechanism in tongue squamous cell carcinoma (TSCC). We found that OA effectively inhibited TSCC cell proliferation in a dose- and time-dependent manner. OA treatment in TSCC significantly induced cell cycle G0/G1 arrest, increased the proportion of apoptotic cells, decreased the expression of CyclinD1 and Bcl-2, and increased the expression of p53 and cleaved caspase-3. OA also obviously induced the formation of autolysosomes and decreased the expression of p62 and the ratio of LC3 I/LC3 II. The expression of p-Akt, p-mTOR, p-S6K, p-4E-BP1 and p-ERK1/2 was significantly decreased in TSCC cells after treatment with OA. Moreover, tumor growth was significantly inhibited after OA treatment in a xenograft mouse model. The above results indicate that OA has a potent anticancer effect in TSCC by inducing apoptosis and autophagy via blocking the Akt/mTOR pathway. Thus, OA is a potential TSCC drug that is worthy of further research and development.

## Introduction

Traditional Chinese medicine (TCM) has been proven to have anti-tumor effects on many types of cancer. TCM herbal treatment has also been shown to increase chemotherapy efficiency, reduce toxicity, prolong survival time, and improve immune functions^1–5^. Brucea javanica oil (BJO) is extracted from the seeds of the herb medicine Brucea javanica, and its main active component is oleic acid (OA)^6^. OA has also attracted much attention in the “Mediterranean diet”, characterized by a high olive oil (rich in OA) consumption^7^. BJO or OA has shown anticancer effects in many types of cancers, such as prostate, breast and colorectal cancer, and OA is commonly administered in combination with chemotherapy^6, 8–11^.

Several mechanisms have been proposed for the antiproliferative effect of OA. Moon *et al*. illustrated that OA could cross-regulate the AMPK/S6 axis and up-regulate tumor suppressor genes (p53, p21, and p27) in esophageal cancer cells^12^. Fu *et al*. also found that OA led to a production of reactive oxygen species (ROS) and up-regulation of NOX4 protein^13^. In short, the effects of OA on cancer cells include effects on the cell membrane, apoptosis, autophagy, mitochondria, proteasome inhibition, cell adhesion and glycolysis^14, 15^.

Despite advances in surgical techniques, chemotherapy and radiation treatments, approximately 50% of patients with tongue squamous cell carcinoma (TSCC) do not survive for more than 5 years after diagnosis as a result of local recurrence or metastasis of the primary tumor^16^. Recently, numerous studies have indicated that natural remedies or TCM have therapeutic effects on neoplastic diseases^1–5^. Although, both epidemiological and animal studies have reported a protective role of OA in several cancers, the beneficial effects of OA in TSCC remain unknown. Moreover, the mechanisms behind the antitumor effect of OA are not well understood. Askari *et al*. found that the levels of OA in oral squamous cancer cell (OSCC) tissue were lower than those in the adjacent normal-appearing squamous tissue^17^. Thus, lower levels of OA may be related to the development and progression of OSCC. To investigate the anticancer effect of OA on TSCC and the mechanism behind its anticancer effect, we first investigated the anticancer effect of OA on TSCC *in vitro* and *in vivo*, and the cell cycle, apoptosis, autophagy and the Akt/mTOR signaling pathway were further analyzed. We found that OA had significant anticancer effects on TSCC both *in vitro* and *vivo*. OA induced cell cycle G0/G1 arrest, apoptosis, and autophagy by modulation of the Akt/mTOR pathway in the treatment of TSCC.

## Results

### OA inhibits the proliferation of TSCC cells ***in vitro***

**Figure 1:**
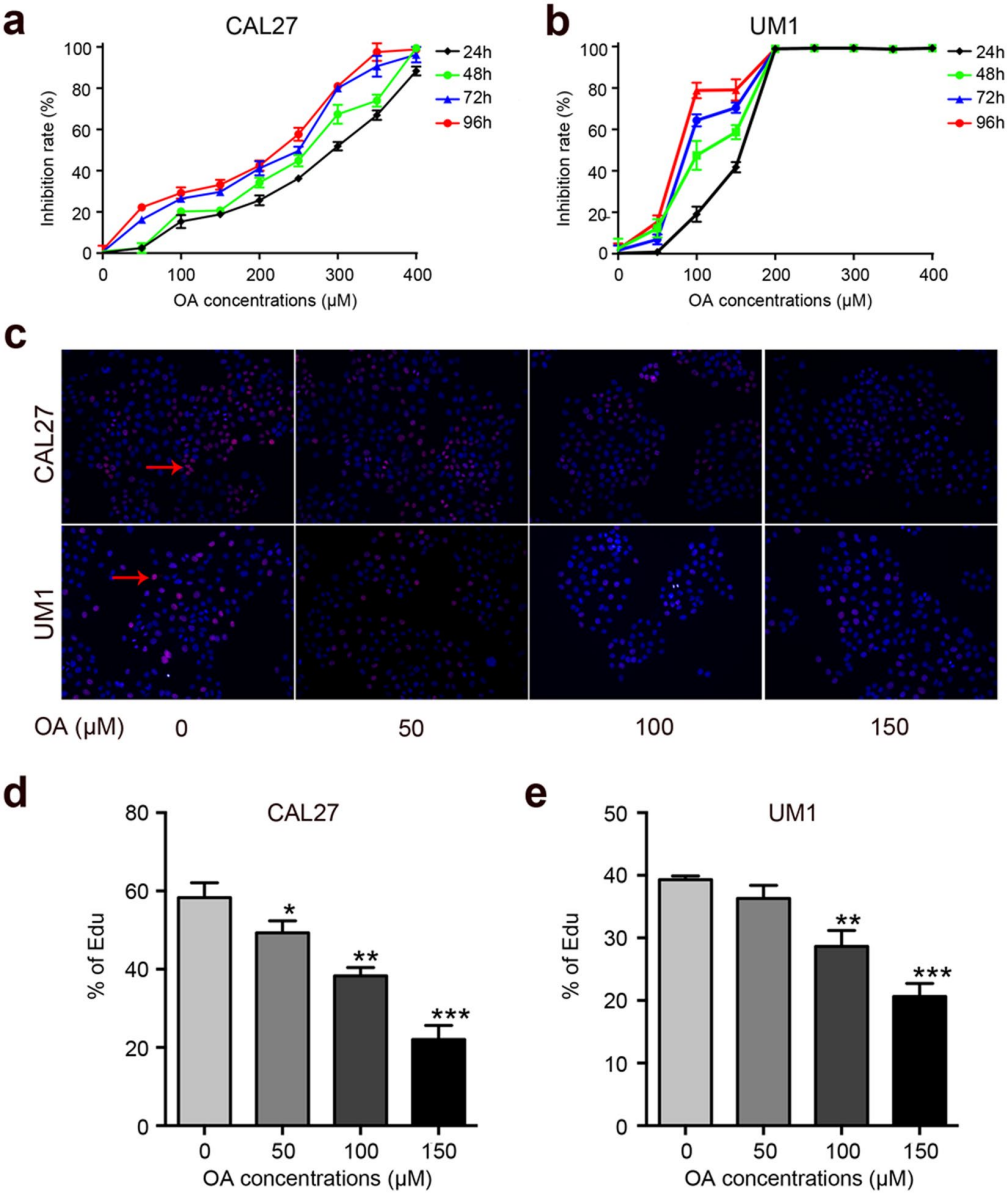
OA inhibits TSCC cells proliferation. (**a**,**b**) TSCC cells (CAL27 and UM1) were treated with OA (0–400 μM) for 24 h, 48 h, 72 h or 96 h, and the inhibition rate of cell proliferation was detected using a Cell Counting kit-8 assay. **(c**–**e)** The capacity of cell proliferation was also tested using an Edu assay after treatment with different concentrations of OA for 24 h. Arrows indicate the proliferating cells. Data are the mean ± standard deviation of three independent experiments (*p < 0.05, **p < 0.01, ***p < 0.001).

First, we performed a CCK8 assay to examine the effect of OA on the proliferation of TSCC cells (CAL27, UM1 cells). As shown in Fig. 1a-b, OA significantly inhibited the proliferation of TSCC cells. The anti-proliferation effect of OA on TSCC cells was portrayed in a dose- and time-dependent manner. The IC50 values of OA were 291~228 μM and 159~78 μM for CAL27 and UM1 cells, respectively (Table 1). The population doubling times of TSCC cell lines were prolonged after treatment with OA, from 69.1 h to 126.8 h for CAL27 cells and from 67 h to 90.7 h for UM1 cells (Table 2). Based on these results, we chose four concentrations (0, 50, 100, and 150 μM) that were below the IC50 at 24 h treatment for further experiments.

**Table 1:** The IC50 of OA in the treatment of TSCC.

Time (h)	IC50 (μM)	
	CAL27	UM1
24	291	159
48	266	112
72	253	89
96	228	78

**Table 2:** The population doubling time of TSCC cells with OA treatment.

OA (μM)	Population doubling time (h)	
	CAL27	UM1
0	69.1	67
50	100.8	90.7
100	116.1	—
150	124.2	—
200	126.8	—
>250	—	—

Moreover, the proliferation abilities of TSCC cells were also investigated using an Edu assay. As shown in Fig. 1c-e, the proliferation of TSCC cells was significantly inhibited at the concentrations of 100 and 150 μM. These results indicated that OA can inhibit the proliferation of TSCC cells.

### OA induces cell cycle G0/G1 arrest in TSCC cells

**Figure 2:**
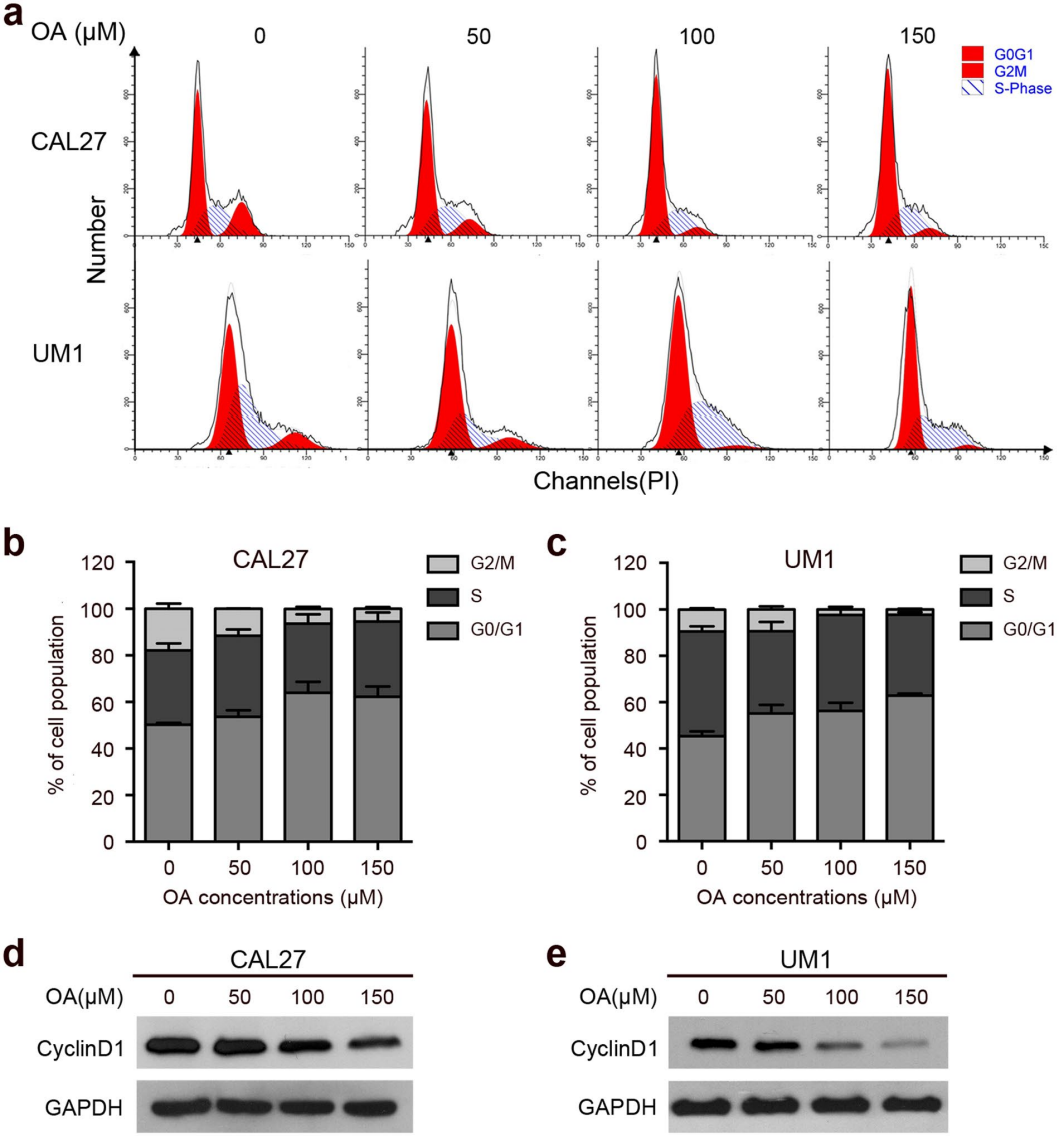
OA induces cell cycle arrest in the G0/G1 phase in TSCC cells. The TSCC cells were treated with different concentrations of OA (0, 50, 100, or 150 μM) for 24 h. **(a)** The flow cytometric histograms show the cell cycle arresting effect of OA in CAL27 and UM1 cells. **(b**,**c)** Cell cycle arrest during the G0/G1, S, and G2/M phases was found after treatment with OA. The results are presented as the mean ± standard deviation for three independent experiments. **(d**,**e)** The G0/G1 cell cycle regulator CyclinD1 was detected by Western blot. GAPDH was used as the internal control.

As shown in Fig. 2a-c, TSCC cells were significantly arrested in the G0/G1 phase in a concentration-dependent manner after treatment with OA. Compared to the controls, the percentage of cells in the G0/G1 phase was significantly increased from 50.28 ± 0.78% to 62.21 ± 3.38% in CAL27 cells and from 45.36 ± 1.50% to 62.88 ± 0.59% in UM1 cells. The percentage of cells in the G2/M phase was correspondingly decreased from 17.87 ± 1.57% to 5.41 ± 0.56% in CAL27 cells and from 9.43 ± 0.95% to 2.26 ± 0.30% in UM1 cells after OA treatment. The percentage of cells in the S phase was not obviously changed (Fig. 2b-c)

Moreover, we detected the expression of the key G0/G1 cell cycle regulator CyclinD1 and found that the protein levels of CyclinD1 were markedly decreased after OA treatment in TSCC cells (Fig. 2d-e). These results indicated that OA induced cell cycle G0/G1 arrest in TSCC cells.

### OA induces apoptosis in TSCC cells

**Figure 3:**
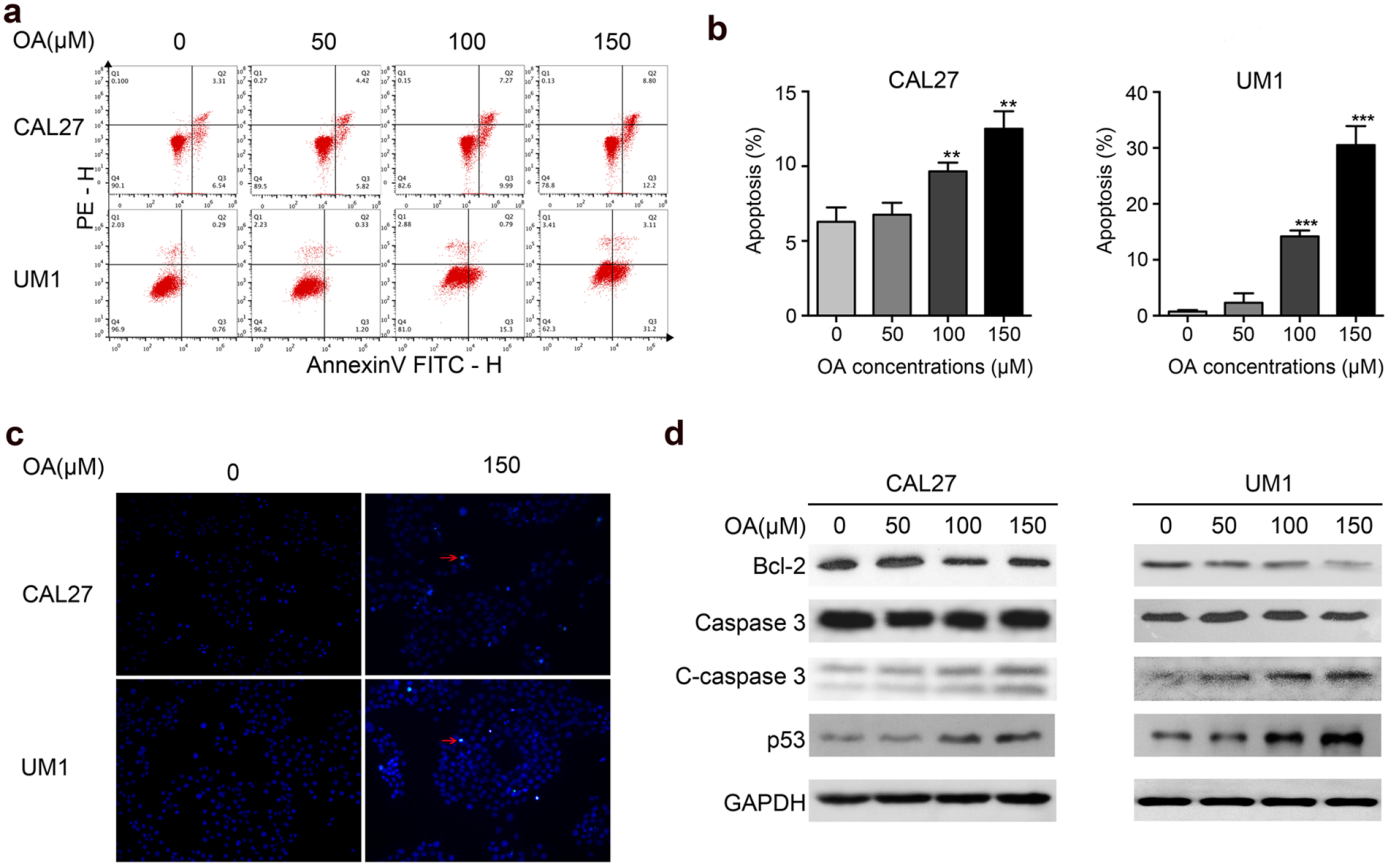
OA induces apoptosis in TSCC cells. The TSCC cells were treated with different concentrations of OA (0, 50, 100, or 150 μM) for 24 h. **(a)** Flow cytometric plots showing OA-induced apoptosis in CAL27 and UM1 cells. The cells in the lower right corner represent early apoptosis, and the top right corner shows the dead cells. **(b)** Bar graphs showing that 24 h OA treatment induced a marked apoptotic cell death in CAL27 and UM1 cells. Data are the mean ± standard deviation of three independent experiments (**p < 0.01, ***p < 0.001). **(c)** TUNEL assay showing OA-induced apoptosis of CAL27 and UM1 cells after treatment with OA. Arrows indicate apoptotic cells. **(d)** The expression of apoptosis-related proteins (Bcl-2, caspase-3, cleaved-caspase-3 and p53) was detected by Western blot. GAPDH was used as the internal control.

To further investigate how OA inhibits cell proliferation in TSCC cells, CAL27 and UM1 cells were treated with OA (0, 50, 100, or 150 μM) for 24 h. As shown in Fig. 3a, the percentages of TSCC cells undergoing early apoptosis were significantly increased in a concentration-dependent manner after treatment with OA. The proportion of apoptotic cells was up to 12.2 ± 1.05% for CAL27 cells and up to 31.2 ± 3.1% for UM1 cells after treatment with OA. Apoptosis was also investigated with TUNEL staining. As shown in Fig. 3c, OA obviously increased apoptosis in TSCC cells. Moreover, we discovered that OA treatment obviously inhibited the expression of the anti-apoptotic protein Bcl-2 and enhanced the expression of the pro-apoptotic proteins cleaved caspase-3 and p53 in CAL27 and UM1 cells (Fig. 3d).

### OA induces autophagy in TSCC cells

**Figure 4:**
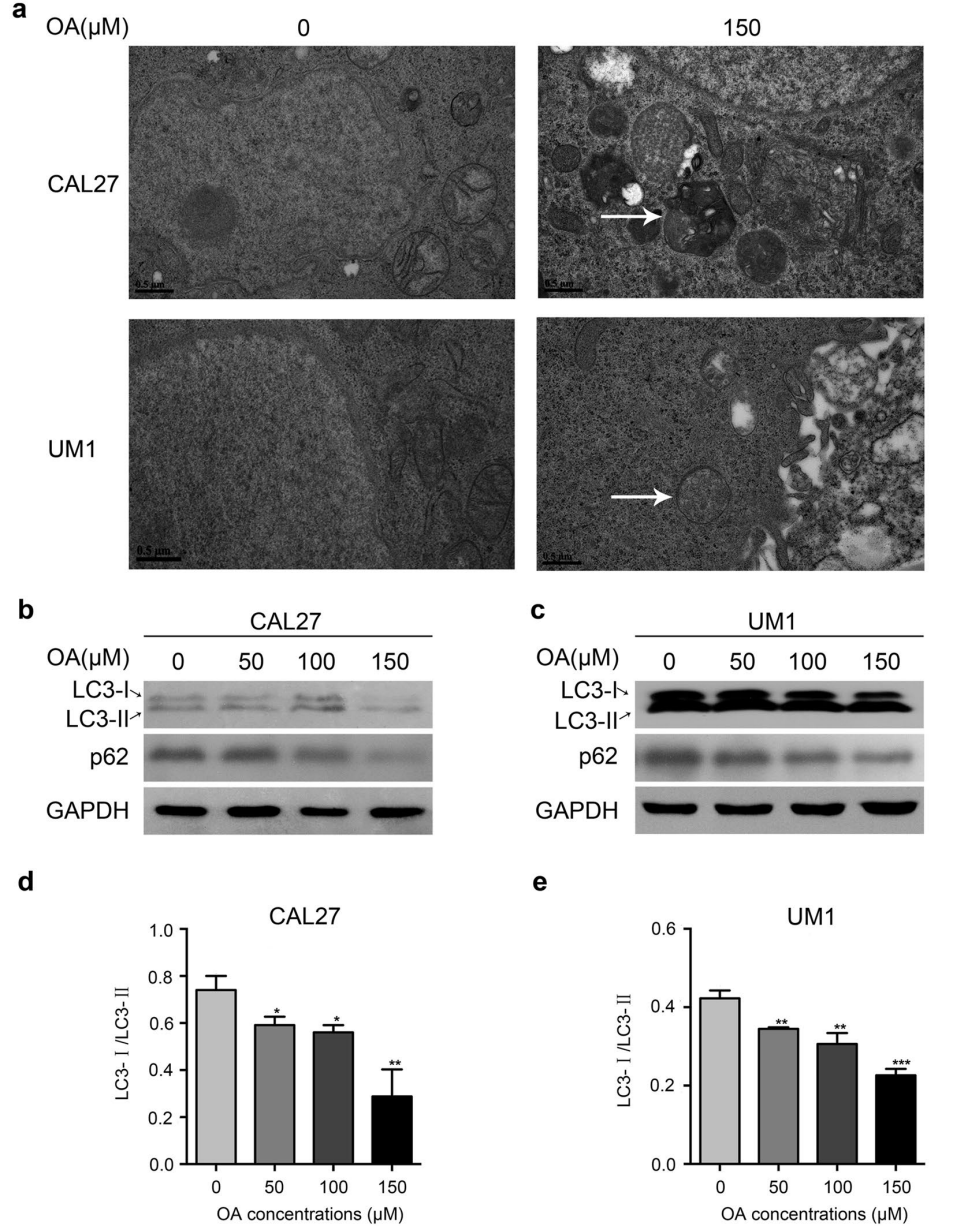
OA induces autophagy in TSCC cells. **(a)** OA-induced autolysosome formation in CAL27 and UM1 cells was observed under a TEM after the TSCC cells were treated with different concentrations of OA (0 or 150 μM) for 24 h. Arrows indicate autolysosomes. **(b**,**c)** Western blot displaying the expression levels of LC3, p62 in TSCC cells after treatment with OA for 24 h. GAPDH was used as the internal control. **(d**,**e)** LC3 protein levels from the above Western blot were quantitated, and the ratio between LC3 I (16 kDa) and LC3 II (14 kDa) was calculated. OA induced conversion of the LC3 protein from LC3 I to LC3 II. Bar graphs showing the LC3 I/LC3 II ratio was significantly decreased after treatment with OA. Data are the mean ± standard deviation of three independent experiments (*p < 0.05, **p < 0.01, ***p < 0.001).

To investigate whether OA induced autophagy in the TSCC cells, transmission electron microscopy (TEM) was used to observe autolysosomes after treatment with OA (0 or 150 μM). As shown in Fig. 4a, OA treatment obviously increased the presence of autolysosome formation in CAL27 and UM1 cells. Autophagy-specific proteins including LC3 and p62 were also detected to evaluate autophagy activity (Fig. 4b-c). After treatment with OA, the expression ratio of LC3-I/ LC3-II was significantly decreased, which means that LC3-I was converted into LC3-II (Fig. 4d-e). The expression of p62 was also obviously decreased after treatment with OA (Fig. 4b-c). These data indicated that OA induced autophagy in CAL27 and UM1 cells.

### OA inhibits the Akt/mTOR signaling pathway

**Figure 5:**
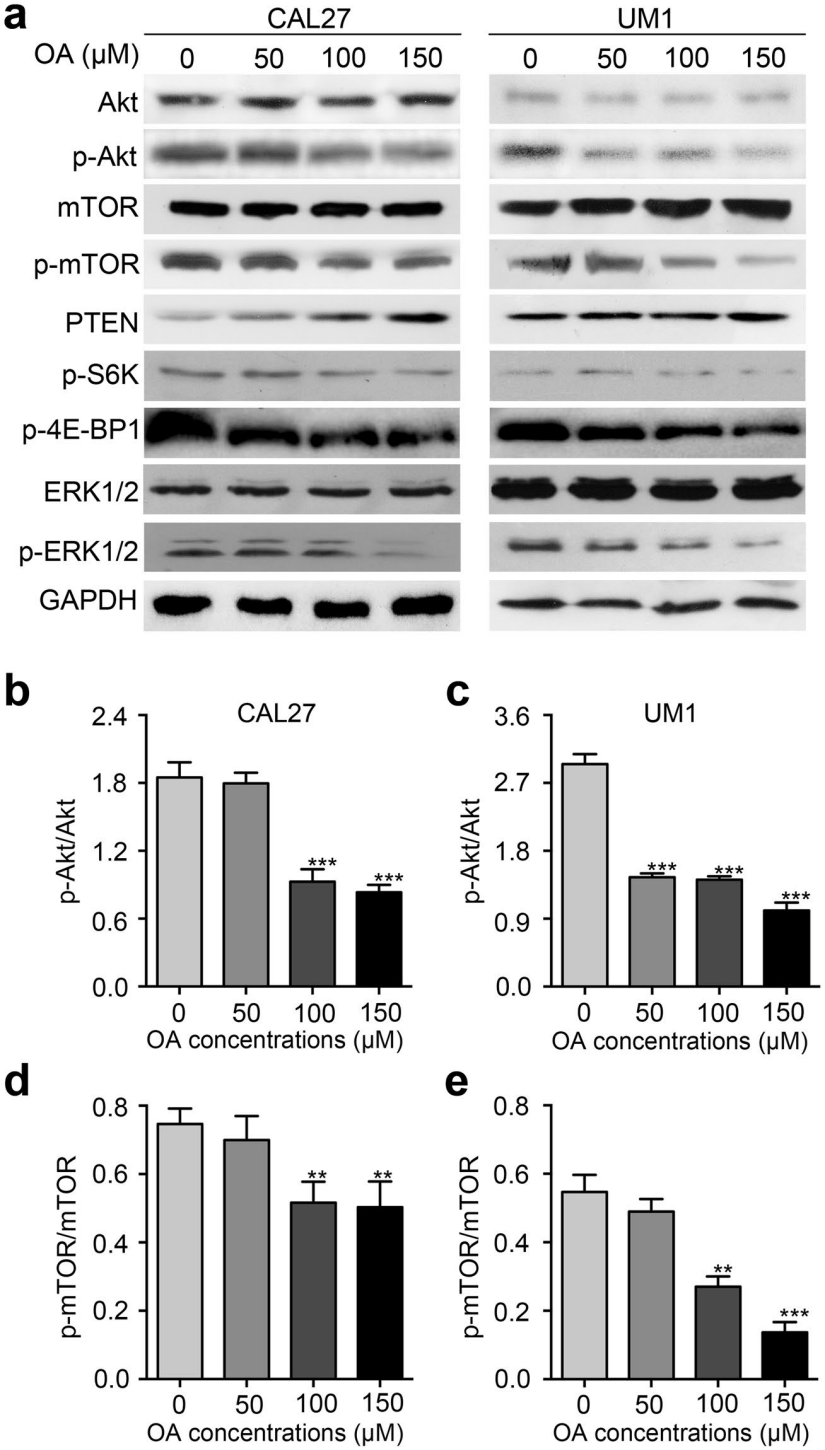
OA inhibits the Akt/mTOR signaling pathway. **(a)** Western blot displaying the expression levels of Akt, p-Akt, mTOR, p-mTOR, PTEN, p-S6K, p-4E-BP1, p-ERK1/2, and ERK1/2 in CAL27 and UM1 cells after treatment with OA for 24 h. GAPDH was used as the internal control. **(b–e)** The protein expression levels from the above Western blot were quantitated. Bar graphs showed that the p-Akt/Akt ratio and p-mTOR/mTOR ratio were significantly decreased after treatment with OA. OA induced a decreased in p-Akt protein expression from 2.1-fold to 0.9-fold and from 3.2-fold to 1.2-fold in CAL27 and UM1 cells, respectively. p-mTOR expression was decreased from 0.78-fold to 0.59-fold in CAL27 cells and from 0.6-fold to 0.2-fold in UM1 cells. The results are presented as the mean ± standard deviation for three independent experiments (**p < 0.01, ***p < 0.001).

To further confirm the possible mechanism of OA-mediated antitumor activity in TSCC, Akt/mTOR pathway-related proteins were detected by Western blot. We found that the expression levels of p-Akt, p-mTOR, p-S6K, p-4E-BP1 and p-ERK1/2 were significantly inhibited by OA in a dose-dependent manner in CAL27 and UM1 cells (Fig. 5a and Fig. S1). Moreover, the protein level of PTEN was increased with the increasing concentrations of OA. The expression of Akt, mTOR, and ERK1/2 was not obviously changed in CAL27 or UM1 cells after treatment with OA (Fig. 5a and Fig. S1).

### OA inhibits the growth of TSCC xenograft tumors ***in vivo***

**Figure 6:**
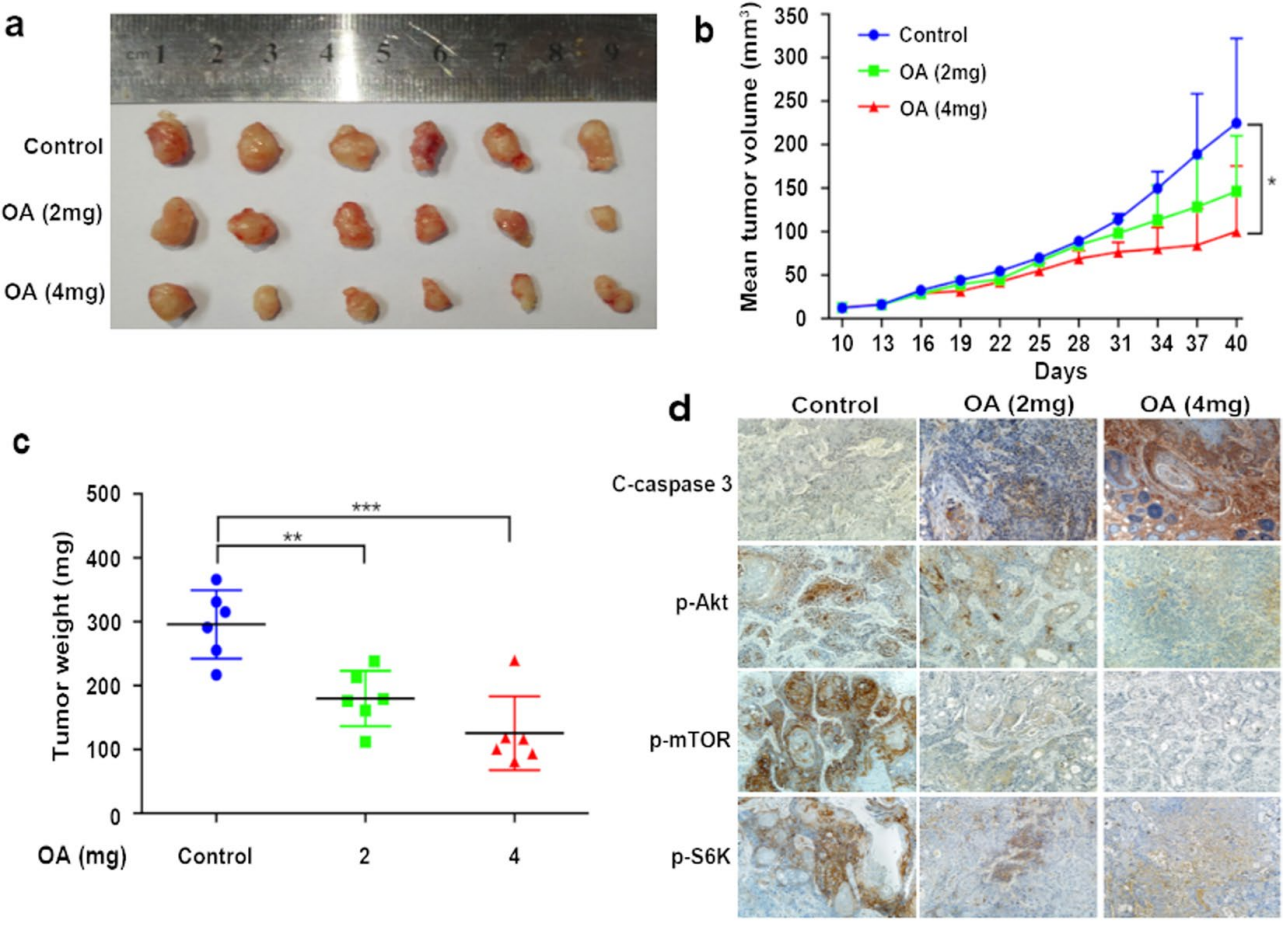
OA inhibited the growth of CAL27 xenograft tumors in nude mice. CAL27 xenograft tumors were treated with OA (2 or 4 mg/kg, i.p, every two days) or (0.2 ml saline as control) for 14 times, and the animals were euthanized on day 40. **(a)** Representative photographs of the gross tumors from nude mice. **(b)** Graphs represent the average tumor volumes of CAL27 xenografts in mice from the control and OA-treated groups. **(c)** Graphs represent the average weights of tumors from the control and OA-treated groups (*p < 0.05, **p < 0.01). **(d)** The expression of p-Akt, p-mTOR, p-S6K, and the cleavage of caspase-3 was detected by immunohistochemical staining and the expression of p-Akt, p-mTOR, and p-S6K was decreased and cleaved-caspase-3 expression was induced after OA treatment.

To investigate whether OA inhibited tumor growth *in vivo*, we chose the CAL27 cell line to establish xenograft tumors in nude mice. The mice were randomly assigned into three groups (0, 2 mg, and 4 mg) when the tumor volume was 12.7 ± 1.7 mm^3^. As shown in Fig. 6, the group treated with OA showed a marked inhibitory action on tumor growth compared with the control group. The tumor inhibition rates on day 40 were 33.3% for the 2-mg group and 58.2% for the 4-mg group, according to the tumor volume (Fig. 6b). The doubling time of the xenograft tumors treated with 0 mg, 2 mg, and 4 mg OA were 7, 9, and 11 days, respectively. The mean weights of the excised tumors decreased by approximately 40% for the 2-mg group and by 56.7% for the 4-mg group (Fig. 6c). Immunohistochemical analyses also showed that OA strongly inhibited the expression of p-Akt, p-mTOR, and p-S6K and induced cleaved-caspase-3 expression in the xenograft tumors (Fig. 6d).

## Discussion

Although most studies have revealed that OA has strong anticancer effects^18, 19^, some studies found that OA could promote the proliferation, migration, invasion of cancer^20, 21^. For example, Fernanda *et al*. confirmed that 100 μM OA decreased the viability of cells^18^, whereas Navarro *et al*. found that OA promoted migration of breast cancer in a dose-dependent manner (0–400 μM)^21^. Here, we investigated the effects of OA on TSCC cells and found that OA had a strong antiproliferation effect and prolonged the cell population doubling time *in vitro* and had a significant anticancer effect in the TSCC xenograft mouse model. Most chemotherapeutic agents induce cell cycle arrest to control cell proliferation, invasion and metastasis^22^. The progression of cells through the cell cycle is under positive control by a series of specific cyclin/CDK complexes and is negatively controlled by specific CKIs (CDK inhibitors)^16^. Many studies have found that G1/S progression is highly regulated by CyclinD1, and the loss of CyclinD1 can induce G1 phase arrest^23–25^. Similarly, we found that the treatment of TSCC cells with OA resulted in a dose-dependent cell cycle arrest in the G0/G1 phase. OA induced CyclinD1 downregulation in TSCC cells. These results indicate that OA suppresses TSCC cell proliferation by G0/G1 phase arrest.

G1-phase cell cycle arrest creates an opportunity for cells to either undergo repair or enter the programmed cell death pathway. There are three forms of programmed cell death (PCD), including apoptosis (type I PCD), autophagy (type II PCD), and programmed necrosis. Many studies have demonstrated that these types of cell death may be triggered by common upstream signals and thus affect cancer development and therapy^26, 27^. Recently, OA was found to trigger autophagic or apoptotic tumor cell death in cancer treatment^28–31^. We showed that OA not only induces autophagy in TSCC cells but also induces apoptosis. During autophagy, the cytoplasm components or organelles that are determined for degradation are conveyed to double-membrane vesicles, known as autophagosomes, which then progress to autolysosomes through the fusion of acidic lysosomes with autophagosomes^32^. In the present study, autolysosomes were observed after treatment with OA, and we also found that LC3-I was converted to LC3-II. LC3 is important for autophagosome formation and function, and LC3 is processed from LC3-I to LC3-II during autophagy^33, 34^. p62, another marker of autophagy, can be incorporated into completed autophagosomes and degraded in autolysosomes^2^ and was also decreased after OA treatment. These data indicate that OA induces autophagy in TSCC. In our study, we also found that OA treatment induces cleavage of caspase-3 *in vitro* and *in vivo* and decreases the expression level of Bcl-2; both are markers of apoptosis^35^. Moreover, the increase of p53 expression was also found after OA treatment; p53 is an important pro-apoptotic factor and can promote apoptosis by activating a number of positive regulators of apoptosis^36^. All these results indicate that OA effectively induces TSCC cell apoptosis. Thus, OA treatment induces TSCC cell G0/G1 arrest and subsequently leads to autophagy and apoptosis.

Apoptosis and autophagy share several common transcriptional regulators and kinase signaling pathways that mediate cell fate^37^. Akt/mTOR is one of them and is well known as the key regulator in a series of cell processes, including metabolism, cell proliferation, and survival^33^. Many reports had revealed that extracts from Chinese medicine have anticancer effects by inducing autophagy, apoptosis, and G0/G1 cell cycle arrest by suppressing the AKT/mTOR signaling pathway^38, 39^. In our study, we also found that OA induced G0/G1 arrest, autophagy and apoptosis and significantly decreased the expression of p-Akt and p-mTOR, p-S6K, p-4E-BP1 in TSCC cells, which means that OA may block the Akt/mTOR signaling pathway. Moreover, OA inhibited the expression of p-Akt, p-mTOR and p-S6K *in vivo*. According to Qi’s research, Akt is known to increase CyclinD1 through inactivation of GSK-3β^24^. Thus, the decreased expression of CyclinD1 and the G0/G1 arrest in TSCC cells after OA treatment may be related to the decreased expression of p-Akt. Mester *et al*. found that the increase in PTEN expression inhibited the Akt/mTOR signaling pathway, leading to cell death and growth regulation^40^. Therefore, in this study, the increased expression of PTEN after treatment with OA in TSCC cells may lead to a decrease in phosphorylation of Akt and mTOR. Moreover, Liu *et al*. found that p53 exert its effect through negative regulation of mTOR to induce apoptosis and autophagy in response to DNA damaging agents^36^. In the present study, we found that p53 expression was induced after treatment with OA in TSCC cells; this may also lead to subsequent autophagy, apoptosis and a decrease in p-Akt and p-mTOR expression. Many recent reports regarding the interaction of the MEK/ERK1/2 and PI3K/Akt/mTOR pathways have suggested that blocking one pathway may also block the other^41^. In our study, we also found that p-ERK1/2 expression was inhibited after OA treatment.

In summary, our results show that OA has valuable anticancer effects on TSCC *in vitro* and *in vivo* through cell cycle G0/G1 arrest and induction of cell death via autophagy and apoptosis. Based on these findings, we conclude that OA may potentially serve as a therapeutic agent for TSCC.

## Materials and Methods

### Cell culture and materials

OA was purchased from Sigma (Sigma-Aldrich, MO, USA) and dissolved in 0.1% NaOH and 10% delipidated bovine serum albumin (d-BSA). OA was prepared as a 10-mM stock solution and stored at −20 °C. BSA was used as a vehicle control. The final concentration of BSA was <0.5%.

TSCC cells (UM1 and CAL27) were cultured in DMEM/F12 medium (UM1) and DMEM medium (CAL27) supplemented with 10% heat-inactivated FBS. Cells were maintained at 37 °C in a 5% CO_2_/95% air humidified incubator. According to most studies, TSCC cells treated with OA were cultured in serum-free medium.

### Cell proliferation detected by CCK8 assay

Cell proliferation was evaluated using a modified Cell Counting Kit-8 (CCK8) assay (Fanbo, Beijing, China) according to the manufacturer’s instructions^42^. In brief, cells were seeded in 96-well plates at a density of 3 × 10^3^ cells per well, and after incubation for 24 h, cells were treated with different concentrations of OA for 24 h, 48 h, 72 h or 96 h. Then, 10 µl of the CCK-8 solution was added to each well of the plate, and the plate was incubated for 2 h in an incubator. The absorbance (optical density, OD) value at 450 nm was determined using a plate reader, and the cell inhibition rate was calculated as follows: [1 − (A_treated _ − A_blank_)/(A_control_ − A_blank_)] × 100%. The population doubling times were calculated as Td = Δt × lg2/(lgN_t_ − lgN_0_), where N_t_ = the OD value at 96 h and N_0_ = the OD value at 24 h.

### Cell proliferation detected by Edu assay

The TSCC cells were treated with different concentrations of OA (0, 50, 100, or 150 μM) for 24 h. After being washed with PBS, cells were incubated in serum-free DMEM containing 10 μmol/L Edu (RiboBio, Guangzhou, China) for 2 h. Then, cells were fixed and stained with Apollo (red) and Hoechst (blue) according to the manufacturer’s instructions. The cells were examined with a fluorescence microscope. The positive cells were identified by red staining, and the numbers of proliferating cells in ten different fields were counted.

### Cell cycle distribution analysis

The effect of OA on cell cycle distribution was determined by flow cytometry as previously described^16^. Briefly, TSCC cells were treated with OA at a concentration of 0, 50, 100, or 150 μM for 24 h. After treatment with OA, cells were washed with D-PBS, centrifuged, and fixed in 70% ethanol at 4 °C overnight. Then, following two washes in PBS, cells were resuspended in 1 ml of PBS containing 20 μg/mL RNase A and 50 μg/mL PI. After 30 min of incubation in the dark at room temperature, a total of 10,000 cells were subject to cell cycle analysis using a flow cytometer. The experiment was performed at least three times with consistent results and analyzed using ModFit software.

### Cellular apoptosis was detected by Flow cytometry and TUNEL assay

The effect of OA on cell apoptosis was evaluated using the Annexin V: PE apoptosis detection kit. Briefly, the cells were collected after 24 h of OA treatment and resuspended in 1 × binding buffer with 5 μL Annexin V: PE and 5 μL 7-amino-actinomycin D (7-AAD) at 1 × 10^5^ cells/mL in a total volume of 150 μL. The cells were incubated in the dark for 15 min at room temperature. An aliquot of 1 × binding buffer (100 μL) was then added to each tube, and the number of apoptotic cells was quantified using a flow cytometer, which collected 10,000 events for analysis. The cells in the lower right corner represent early apoptosis, and the top right corner shows the dead cells.

TUNEL assay was also used to detect apoptosis according to the manufacturer’s instructions (Roche Molecular Biochemicals, Indianapolis, IN). Briefly, cells were plated in 6-well plates and cultured to 80% confluence and then incubated with OA (150 μM) for 24 h. Apoptotic cells were labeled (1 h, 37 °C) with 20 μl TUNEL reaction mixture in the dark after fixation (4% paraformaldehyde) and permeabilization (Triton X-100 0.1% in PBS). Then, cells were stained with DAPI solution in the dark and images were captured under a light microscope.

### Transmission electron microscopy (TEM) analysis

TEM was used to detect autolysosomes (autophagy) after treatment with OA (0 or 150 μM) for 24 h. Treated TSCC cells were collected and fixed in 2.5% glutaraldehyde (Sigma Co., Ltd., USA) for 24 h, washed with 0.1 M PBS (pH 7.4), post-fixed in 1% osmium tetroxide (Polyscience Co., Ltd., USA), and subsequently dehydrated in increasing concentrations of alcohol. The samples were then impregnated with propylene oxide (Merck-Schuchardt Inc., Hohenbrunn, Germany) and embedded in epoxy resin embedding media. After light microscopy examination of semi-thin sections and staining with 2% (w/v) uranyl acetate and 1% (w/v) lead citrate, samples were observed with a JEM-1200EX II (Jeol, Japan) transmission electron microscope. TEM images at 18500x magnification are shown.

### Western blot analysis

Western blot analysis was performed as described previously^43^ to detect the expression levels of CyclinD1, p53, Bcl-2, caspase-3, cleaved-caspase-3, ERK1/2, p-ERK1/2, LC3, p62, PTEN, Akt, p-Akt, mTOR, p-mTOR, p-S6K, p-4E-BP1 and GAPDH (Cell Signaling Technology, MA, USA) in TSCC cells. GAPDH was used as the internal control. The intensity of each band was quantified using the image analyzing computer software Quantity One (Bio-Rad software).

### TSCC xenografts in Nude mice

To investigate the anticancer effect of OA *in vivo*, CAL27 cells (1 × 10^7^/0.2 ml) were subcutaneously injected into the right flanks of 4-wk-old male BALB/c nude mice (purchased from Beijing Vital River Laboratory Animal Technology Co., Ltd)^44^. Ten days later, each xenograft was identifiable as a mass of more than 3 mm in maximal diameter in all groups. Then, mice were randomly assigned into 3 groups (control, 2-mg treated group, 4-mg treated group, n = 6/group). The OA-treated groups were injected intraperitoneally (i.p.) at different concentrations (2 or 4 mg/kg body weight) every two days for 14 times, whereas the control group received saline (0.2 ml) as vehicle. In addition, the animals were euthanized on the 40^th^ day. During this period, all mice were examined every 3 days to assess their health and any evidence of drug toxicity. All the mice appeared to be healthy and there were no obvious signs or symptoms of drug toxicity or loss of body weight during the experimental period. Tumors were measured every 3 days with a standard caliper and the tumor volumes were calculated as ½length × width^2^, and a tumor growth curve (y = Ae^kday^) and the tumor doubling time (ln2/k) were obtained as previously described^44^. At the end of the experiments, tumors were weighed after being separated from the surrounding muscles and dermis.

Tumor samples were fixed in 4% paraformaldehyde and embedded in paraffin wax. Sample sections were dewaxed, rehydrated and stained using Mayer’s hematoxylin and eosin Y solution. Immunohistochemistry (IHC) was used to detect the expression of p-Akt, p-mTOR, p-S6K and cleaved-caspase-3 in xenograft sections.

### Ethics statement

The animal study was approved by the Ethics Committee of the First Affiliated Hospital at Sun Yat-Sen University (2016047). All the methods were carried out in accordance with the approved guidelines.

### Statistical analysis

The results are presented as the mean ± standard deviation for three independent experiments. All statistical analyses were performed using the Statistical Package for the Social Sciences (SPSS, Chicago, IL), Version 19.0. The data were analyzed with a one-way analysis of variance to calculate significance. The value *P* < 0.05 was considered statistically significant.

## Electronic supplementary material

Original data of blots

Supplementary Figure S1-revised
