# Supplementary material for: Oleic acid induces apoptosis and autophagy in the treatment of Tongue Squamous cell carcinomas
Source: Sci Rep. 2017 Sep 12;7:11277. doi: 10.1038/s41598-017-11842-5 (PMC5595908; doi:10.1038/s41598-017-11842-5)

Oleic acid induces apoptosis and autophagy in the treatment of Tongue Squamous cell carcinomas

Lin Jiang[1,2,3 #], Wei Wang [1,2 #], Qianting He [1], Yuan Wu [2], Zhiyuan Lu [1], Jingjing Sun [1], Zhonghua Liu [1], Yisen Shao [2,3 *], Anxun Wang[1 *]

1. Department of Oral and Maxillofacial Surgery, First Affiliated Hospital, Sun Yat-Sen University, Guangzhou, Guangdong, 510080, China

2. Department of Oral and Maxillofacial Surgery, Affiliated Hospital of Jiangxi University of Traditional Chinese Medicine, Nanchang, Jiangxi Province 330006, China

3. School of Stomatology, Nanchang University, Nanchang, Jiangxi Province 330006, China

# These authors contributed equally to this work.

*: ***Correspondence author:*** Anxun Wang, ***email:*** wang_anxun@aliyun.com；*Yisen Shao*, ***email:*** yshshao@163.com

Original data of Fig.2 d-e

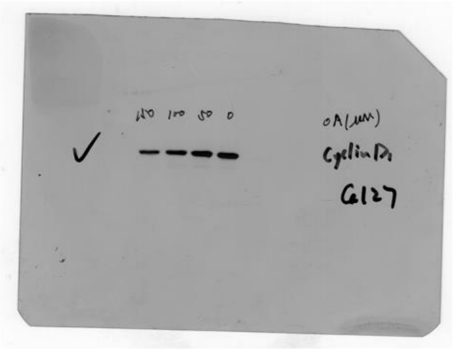

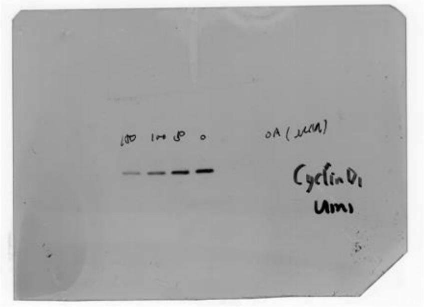

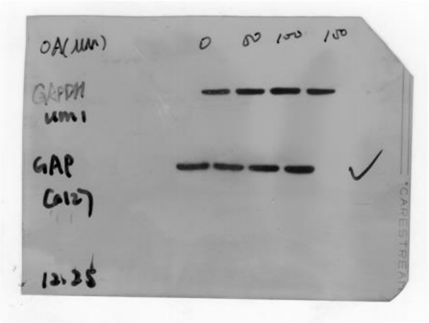

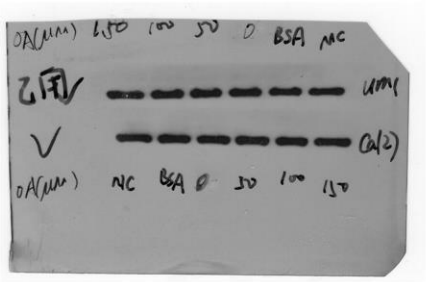

Original data of Fig. 3 d

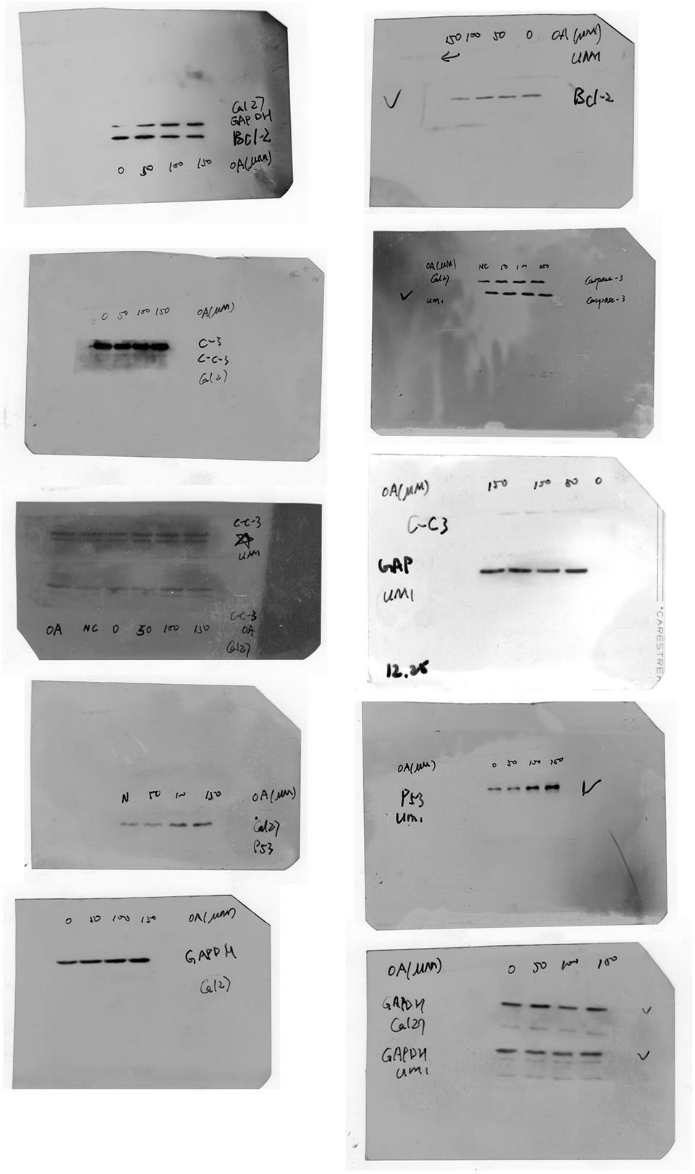

Original data of Fig.4 b-c

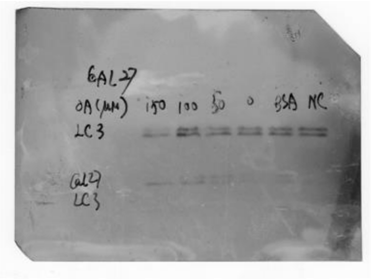

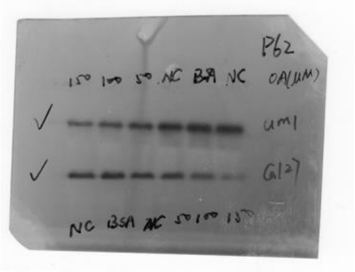

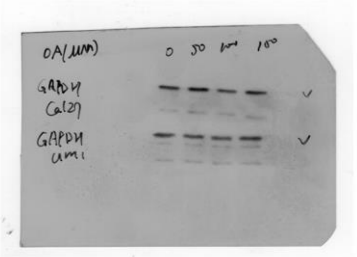

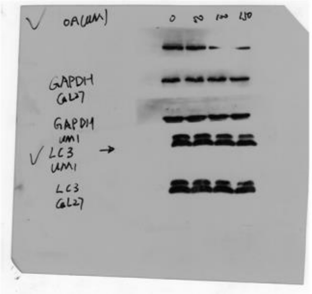

Blots of CAL27 in Fig.5 a

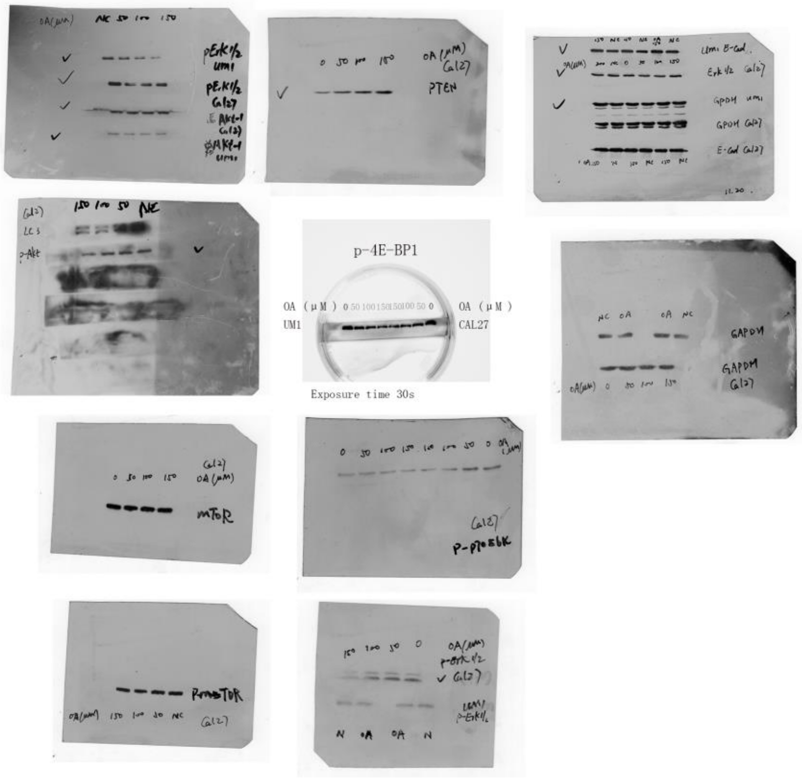

Blots of UM1 in Fig.5 a

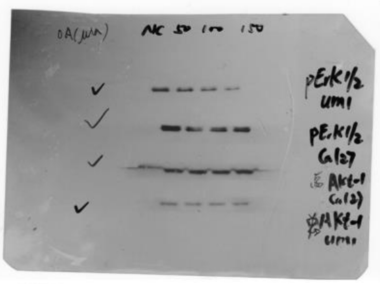

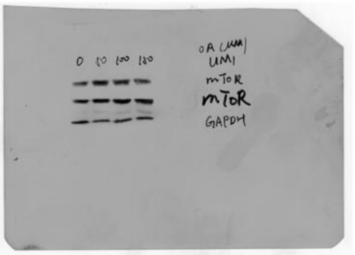

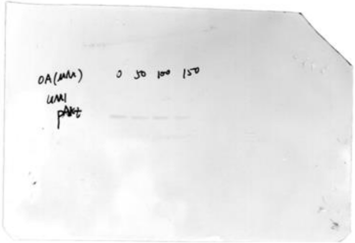

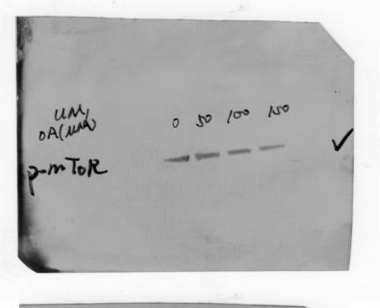

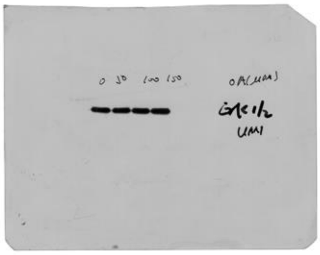

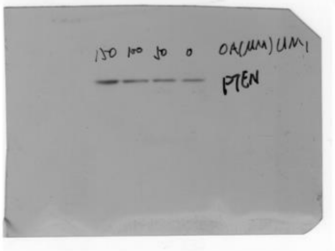

p-4E-BP1

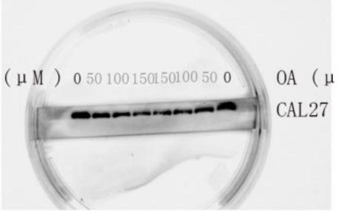

Exposure time 30s

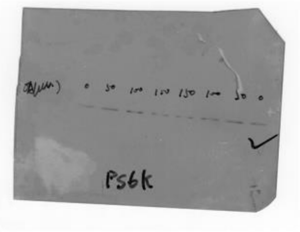

Supplement: Supplementary file 1 — Original data of blots [file 41598_2017_11842_MOESM1_ESM.pdf]
