# Supplementary material for: Oleic acid induces apoptosis and autophagy in the treatment of Tongue Squamous cell carcinomas
Source: Sci Rep. 2017 Sep 12;7:11277. doi: 10.1038/s41598-017-11842-5 (PMC5595908; doi:10.1038/s41598-017-11842-5)
Supplement: Supplementary file 2 — Supplementary Figure S1-revised [file 41598_2017_11842_MOESM2_ESM.pdf]

Oleic acid induces apoptosis and autophagy in the treatment of Tongue Squamous cell carcinomas

Lin Jiang[1,2,3 #], Wei Wang [1,2 #], Qianting He [1], Yuan Wu [2], Zhiyuan Lu [1], Jingjing Sun [1], Zhonghua Liu [1], Yisen Shao [2,3 *], Anxun Wang[1 *]

1. Department of Oral and Maxillofacial Surgery, First Affiliated Hospital, Sun Yat-Sen University, Guangzhou, Guangdong, 510080, China

2. Department of Oral and Maxillofacial Surgery, Affiliated Hospital of Jiangxi University of Traditional Chinese Medicine, Nanchang, Jiangxi Province 330006, China

3. School of Stomatology, Nanchang University, Nanchang, Jiangxi Province 330006, China

# These authors contributed equally to this work.

*: ***Correspondence author:*** *Yisen Shao*, ***email:*** yshshao@163.com; Anxun Wang, ***email:*** wang_anxun@aliyun.com

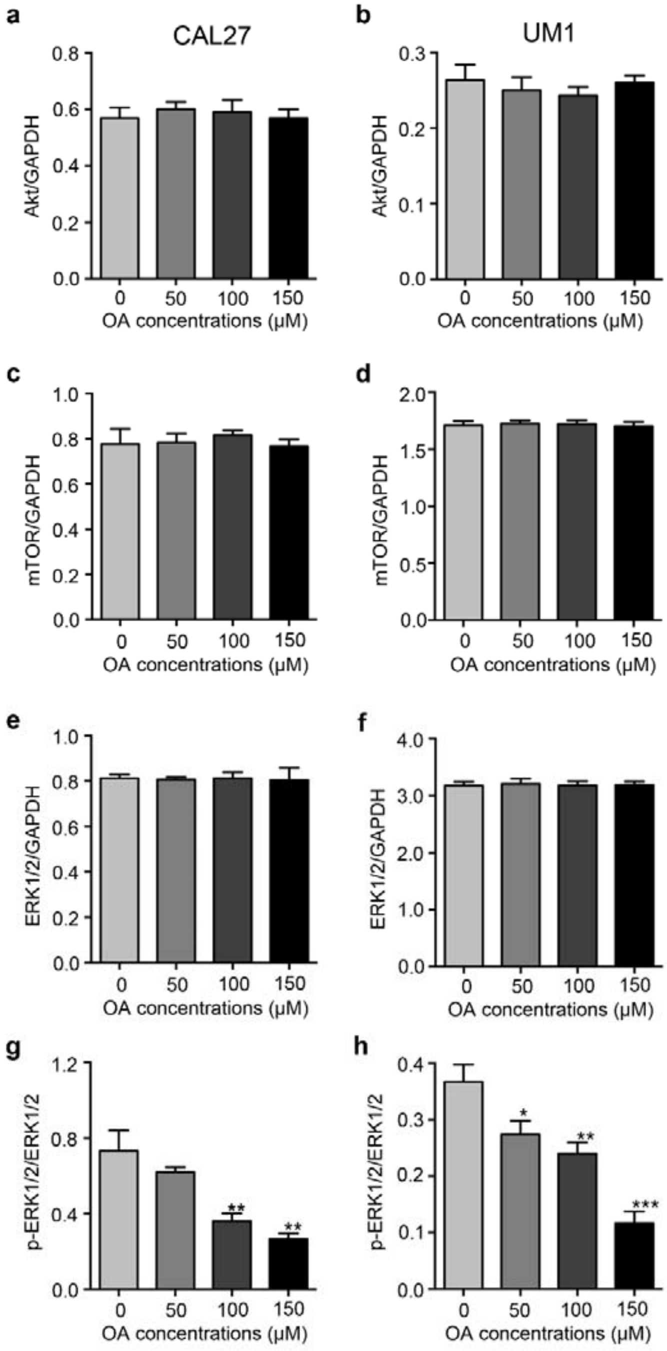

Supplementary Figure S1. The expression of Akt, mTOR, ERK1/2, p-ERK1/2 after treated with OA for 24 h in TSCC cells.

(a-f) The expression level of Akt, mTOR, ERK1/2 showed no obvious change between the OA treated group and the control group. (g-h) The expression of p-ERK1/2 was significantly decreased compared with the control group. (***p<0.001)
